# Knowledge, attitudes and practices of pharmacovigilance in the context of COVID-19 in health professionals of the peruvian social insurance

**DOI:** 10.17843/rpmesp.2022.391.10651

**Published:** 2022-03-31

**Authors:** L. Yesenia Rodríguez-Tanta, Diego André Ale-Mauricio, Violeta Saromo-Meléndez, Anaís Lazarte-Ramos, Elisa Gálvez-Dávila, Gisela Pecho-Arias, Jack Urruchi-Huertas, Paola Fernández-Rojas, Raquel Delgado-Escalante

**Affiliations:** 1 Instituto de Evaluación de Tecnologías en Salud e Investigación, EsSalud, Lima, Peru. Instituto de Evaluación de Tecnologías en Salud e Investigación EsSalud Lima Peru; 2 Instituto Nacional de Salud, Lima, Peru. Instituto Nacional de Salud Lima Peru

**Keywords:** Drug Safety, Pharmacovigilance, Health Knowledge, Attitudes, Practice, Healthcare Workers, COVID-19

## Abstract

The study aimed to evaluate a group of health professionals' knowledge, attitudes, and practices on pharmacovigilance in the context of COVID-19 in the Peruvian Social Health Insurance (EsSalud). A descriptive secondary analysis was carried out on a database that included responses from an online survey conducted by the Institutional Referral Center for Pharmacovigilance and Technovigilance of EsSalud. Of 144 participants, 66% showed a high level of knowledge and 81.2% had a positive attitude; however, 71.5% had an inadequate level of pharmacovigilance practice. Although EsSalud professionals demonstrated a high level of knowledge and positive attitude to implement pharmacovigilance, this is not reflected in the practice of this activity during the SARS-CoV-2 pandemic. Strategies should be implemented to integrate pharmacovigilance into healthcare activities to benefit patient safety.

## INTRODUCTION

Pharmacovigilance is the science and activity related to the detection, evaluation, understanding and prevention of adverse effects of drugs or any related problem that causes unintended harm to the patient; one of its essential components is the reporting of adverse drug reactions (ADRs) ^1^. The Peruvian Pharmacovigilance System has been in place since 2000 and is managed by the General Directorate of Medicines, Supplies and Drugs (DIGEMID). One of the institutions that is part of this system is the Peruvian Social Health Insurance (EsSalud) through the Institutional Referral Center for Pharmacovigilance and Technovigilance (CRI-EsSalud) ^2^.

Despite the fact that pharmacovigilance is part of health care activities, there is little evidence regarding the knowledge and practice of pharmacovigilance by health care professionals in some parts of the world. A recent study estimated that slightly more than half (52.2%) of healthcare personnel had inadequate knowledge of pharmacovigilance ^3^ and up to 86.6% were unaware of the impact of ADR reporting ^4^ ^,^ ^5^. There are healthcare professionals who consider that reporting ADRs is an obligation rather than a healthcare task ^6^; however, it has also been reported that non-prescribing professionals report ADRs more frequently and with less knowledge of pharmacovigilance ^7^.

The role of pharmacovigilance during the COVID-19 pandemic is even more important because therapeutic alternatives have been used without sufficient scientific evidence for the treatment of COVID-19 ^8^ ^-^ ^10^, which could result in more risks than benefits because their safety profiles in this type of patients are unknown. Therefore, this study aims to describe the knowledge, attitudes and practices regarding pharmacovigilance in the context of the COVID-19 pandemic in a group of Peruvian social security health professionals.

**KEY MESSAGES**

Motivation for the study: To determine whether pharmacovigilance activities during the COVID-19 pandemic were understood, accepted and put into practice by Peruvian social security health professionals.

Main findings: Despite the fact that most health professionals recognize the importance of implementing pharmacovigilance and are willing to do so, it is not usually applied during clinical practice.

Implications: This study identifies the problems related to the implementation of pharmacovigilance in the health care setting and proposes strategies to improve patient safety.

## THE STUDY

### Design of the study

A descriptive secondary analysis conducted on a database that contains responses from an online survey conducted by the ESSALUD Institutional Referral Center for Pharmacovigilance and Technovigilance (CRI-ESSALUD) regarding pharmacovigilance knowledge, attitudes and practices in the context of COVID-19, aimed at health professionals during the months of June to August 2020.

Records of EsSalud health personnel were included. Duplicate records and those with at least one missing response in the database were excluded.

### Procedures

In January 2021, we requested authorization from CRI-ESSALUD to access the database with the responses from the pharmacovigilance survey. This database included 156 records and 40 variables distributed in information regarding: a) sociodemographic characteristics b) knowledge c) attitudes and d) pharmacovigilance practices. The database was sorted and the records that met the selection criteria were identified and then analyzed.

### Variables

The sociodemographic information included sex, profession, city and work center, which included the variables “healthcare center” and “service” that was dichotomized into centers with or without inpatient care service, in accordance with what is described in numeral 6.8 about the activities of public and private health facilities of the NTS 123-MINSA/DIGEMID-V.01 “Technical health standard that regulates pharmacovigilance and technovigilance activities of pharmaceutical products, medical devices and sanitary products” ^11^.

The items that measure knowledge, attitudes and practices were standardized in categories and dichotomized as correct and incorrect answers. Scores were assigned to each section by adding them together. Binary responses were assigned a value of 1 if they were correct and 0 if they were incorrect. Likert scale questions were assigned a score of 0 when answered as “never”, 1 “rarely”, 2 “sometimes” and 3 “always”. The overall score for knowledge, attitudes and practices was dichotomized into “low” and “high” considering the median as the cut-off point.

### Statistical analysis

For the univariate descriptive analysis of the categorical variables, we used relative and absolute frequencies. During the bivariate analysis for the comparison of proportions in contingency tables, we used the chi-square test and Fisher’s exact test when more than 20% of the cells had expected values less than 5 or when any expected value was less than 1. To evaluate statistical significance, we considered a p-value of less than 0.05. All analyses were performed with STATA version 15.0.

### Ethical criteria

This study was approved by the Specific Research Ethics Committee for COVID-19 of EsSalud. Likewise, the study was registered in the PRISA platform of the National Institute of Health with the registration code 1F00730F- EA69-4FD2-87B8-391F081B76D6 in strict compliance with current regulations.

## FINDINGS

Out of the 156 records in the database with complete pharmacovigilance survey results, eight were excluded because they were responses from health professionals from institutions other than EsSalud; and four were duplicates. Finally, the records of 144 health professionals were included, of which 63.9% were female, 42.4% were from Lima and the majority were pharmacists (36.1%) and physicians (35.4%) (Table 1).

**Table 1:** Characteristics of health professionals who participated in the survey on knowledge, attitudes and practices of pharmacovigilance in the context of COVID-19.

Characteristics	n (%)
Sex	
Male	52 (36.1)
Female	92 (63.9)
Profession	
Physician	51 (35.4)
Pharmaceutical chemist	52 (36.1)
Nurse	26 (18.1)
Other	15 (10.4)
City	
Lima	61 (42.4)
Arequipa	18 (13.0)
Trujillo	8 (5.6)
Iquitos	7 (4.9)
Other	50 (34.1)
Type of health center	
With inpatient service	111 (77.1)
Without inpatient service	33 (22.9)

**Figure 1:**
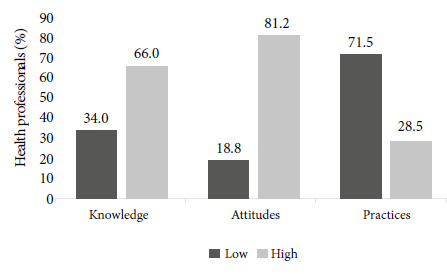
Level of knowledge, attitudes and practices regarding pharmacovigilance in the context of COVID-19 in EsSalud health professionals.

Most had a high level of knowledge about pharmacovigilance (Figure 1); 97.2% adequately defined pharmacovigilance and 78.5% recognized its importance in the identification of new ADRs. Likewise, 81.3% recognized the existence of the Peruvian Pharmacovigilance System, 91% answered that all ADRs (known and unknown) should be reported for drugs used in COVID-19. It is noteworthy that 89.6% considered ivermectin to be an unsafe drug in the treatment of COVID-19 (Table 2). The level of knowledge was dependent on the profession p<0.001, 90% of the pharmacists and 54.9% of the physicians had a high level of knowledge in contrast to other health professionals. The city where the professionals work also showed significant differences (p=0.018), those from Lima and Trujillo had a higher level of knowledge (Table 3).

**Table 2:** Knowledge, attitudes and practices of health professionals on pharmacovigilance in the context of COVID-19 (n=144).

Questions	n	%
Do you know what pharmacovigilance is?		
Yes	140	97.2
Are clinical trials sufficient to know the safety profile of drugs?		
Yes	31	21.5
Do you believe there is a National Pharmacovigilance System?		
Yes	117	81.3
What is an adverse drug reaction (ADR)?		
An adverse effect, i.e., an unintended harmful response to a drug.	144	100
A problem related to drug quality	0	0.0
Doesn’t know	0	0.0
What type of ADRs should be reported for drugs used in COVID-19?		
Known	4	2.8
Not known	9	6.2
Both	131	91
Do you know if EsSalud has considered the monitoring of ADRs in patients receiving COVID-19 pharmacological treatment?		
Yes	76	52.8
Are you familiar with the institution’s ADR notification form (yellow sheet)?		
Yes	105	72.9
Are you familiar with EsSalud’s ADR notification flow?		
Yes	84	58.3
What is the purpose of reporting ADRs to COVID-19 drug treatment?		
Preventing and minimizing patient harm	138	95.8
To comply with a bureaucratic/administrative requirement	5	3.5
Doesn’t know	1	0.7
Do you consider ivermectin to be a safe drug in the treatment of COVID-19?		
Yes, it doesn’t require safety monitoring during use.	15	10.4
No, it requires safety monitoring during use.	129	89.6
In the case of identifying an adverse reaction to the drugs used in the treatment of COVID-19, do you agree to report it?		
Agrees	139	96.5
Doesn’t agree	2	1.4
Doesn’t know	3	2.1
Do you believe that the system for reporting ADRs to the drugs used in COVID-19 benefits the patient?		
Yes	137	95.1
No	2	1.4
Doesn’t know	5	2.1
In the event that you become aware of a suspected ADR to any COVID-19 drug, do you think that reporting it could have legal implications?		
Yes	23	16.0
No	90	62.5
Doesn’t know	31	21.5
Do you consider that reporting a suspected ADR to COVID-19 drugs is a time-consuming activity with no results?		
Yes	32	22.2
No	112	77.8
Have you ever reported an ADR in the adverse drug reaction notification form (yellow sheet) at EsSalud?		
Never	62	43.0
Rarely	39	27.1
Sometimes	20	13.9
Always	23	16.0
Have you identified any ADR to any of the drugs foreseen in the treatment for COVID-19 and have you reported it?		
Yes	15	10.4
No	113	78.5
Doesn’t know	16	11.1
Have you received training on Intensive Pharmacovigilance for COVID-19 drug treatment?		
Yes	17	11.8
No	122	84.7
Doesn’t know	5	3.5
Are you participating in the implementation of intensive pharmacovigilance of COVID-19 drug treatment in EsSalud?		
Yes	21	14.6
No	118	81.9
Doesn’t know	5	3.5
Have you read the safety communications on the proposed drug treatments for COVID-19 issued by IETSI-EsSalud?		
Yes	91	63.2
No	53	36.8
How often do you usually read updates to the safety information in the pharmacological recommendations against COVID-19?		
Never	6	4.2
Rarely	19	13.2
Some times	57	39.6
Always	62	43.0
How often do you check the technical data sheet to ensure the safe use of medicines?		
Never	5	3.5
Rarely	7	4.8
Some times	80	55.6
Always	52	36.1

**Table 3:** Knowledge, attitudes and pharmacovigilance practices of health professionals in the context of COVID-19, according to their characteristics.

Characteristics	Knowledge			Attitudes			Practices		
	Low	High	p-value	Low	High	p-value *	Low	High	p-value
Sex									
Male	23 (44.2)	29 (55.8)	0.052 ^a^	12 (23.1)	40 (76.9)	0.317 ^a^	42 (80.8)	10 (19.2)	0.065 ^a^
Female	26 (28.3)	66 (71.7)		15 (16.3)	77 (83.7)		61 (66.3)	31 (33.7)	
Profession									
Physician	23 (45.1)	28 (54.9)	<0.001 ^a^	12 (23.5)	39 (76.5)	0.204 ^b^	39 (76.5)	12 (23.5)	0.077 ^b^
Pharmacist	4 (7.7)	48 (92.3)		7 (13.5)	45 (86.5)		32 (61.5)	20 (38.5)	
Nurse	12 (46.2)	14 (53.8)		3 (11.5)	23 (88.5)		18 (69.2)	8 (30.8)	
Other	10 (66.7)	5 (33.3)		5 (33.3)	10 (6.7)		14 (93.3)	1 (6.7)	
City									
Lima	23 (37.1)	39 (62.9)	0.013 ^b^	12 (19.4)	50 (80.6)	0.101 b	46 (74.2)	16 (25.8)	0.236 ^b^
Arequipa	11 (61.1)	7 (38.9)		7 (38.9)	11 (61.1)		16 (88.9)	2 (11.1)	
Trujillo	3 (37.5)	5 (62.5)		2 (25.0)	6 (75.0)		6 (75.0)	2 (25.0)	
Iquitos	3 (42.9)	4 (57.1)		0 (0.0)	7 (100.0)		4 (57.1)	3 (42.9)	
Other	9 (18.4)	40 (81.6)		6 (12.2)	43 (87.8)		31 (63.3)	18 (36.7)	
Health center									
With inpatient service	36 (32.4)	75 (67.6)	0.459 ^a^	24 (21.6)	87 (78.4)	0.105 ^a^	74 (66.7)	37 (33.3)	0.018 ^a^
Without inpatient service	13 (39.4)	20 (60.6)		3 (9.1)	30 (90.9)		29 (87.9)	4 (12.1)	

a Chi-square test, b Fisher’s exact test.

Positive attitudes to implement pharmacovigilance in the context of COVID-19 were found in 81.2% of health professionals; and 95.1% recognized that reporting ADRs would benefit the patient. However, 16% considered that reporting ADRs could have legal implications and 22.2% thought that this activity was time-consuming. Furthermore, a high percentage of women had positive attitudes to implement pharmacovigilance in the context of COVID-19 (Table 2).

The level of pharmacovigilance practices in the context of COVID-19 was low in 71.5% of the health professionals; 43% never reported an ADR and 10.4% identified an ADR and reported it to the CRI-EsSalud. Only 11.8% received training and less than 15% implemented intensive monitoring of patients exposed to “off-label” drugs (drugs outside the indications for which it was approved or used in a different way) against COVID-19. Of all the health professionals, 63.2% read the drug safety communications on “off-label” treatments for COVID-19 issued by the CRI- EsSalud and most (55.6%) “sometimes” reviewed the technical data sheet of the drugs approved by the regulatory agencies. Finally, a higher proportion of professionals working in centers without inpatient service had a “low” level of pharmacovigilance practices in the context of COVID-19 as opposed to those working in centers with inpatient service (p=0.018) (Table 3).

## DISCUSSION

We found that most participants had a high level of pharmacovigilance knowledge in the context of COVID-19. One possible explanation is that, since the beginning of the pandemic, the CRI-EsSalud trained the institution’s professionals in the reporting of ADRs, mainly due to the use of drugs that have not been approved for the treatment of COVID-19. Therefore, it is possible that those who responded to the survey are those who received the training or were directly involved with pharmacovigilance activities at the institution, i.e., personnel of the pharmacovigilance committees or frequent ADR reporters.

Pharmacists had the highest level of knowledge about pharmacovigilance, followed by physicians and other professionals. It is possible that this professional group identifies with issues regarding drug safety and receives training from DIGEMID, which is responsible for conducting the Peruvian Pharmacovigilance System. Outside the context of the pandemic, in Peru, the training of pharmacists is focused on pharmacotherapeutic follow-up, an activity that includes detection and reporting of ADRs. In addition, national regulations and the labor law require pharmacist participation in the access and rational use of medicines ^12^ and in the Peruvian Pharmacovigilance and Technovigilance System. The results of studies carried out in other parts of the world are controversial when it comes to the professionals with the greatest knowledge of pharmacovigilance; some studies identify the pharmacist ^13^^,^^14^ and others, the physician ^15^^,^^16^ ^,^ ^17^.

Positive attitudes towards pharmacovigilance were found in 81.2% of the participants. It is important to note that more than 90% considered that reporting ADRs of drugs used in COVID-19 benefits the patient. Therefore, positive attitudes are important to assess the predisposition of health professionals regarding the report ADRs of drugs without sufficient scientific evidence of their safety and efficacy against SARS-CoV-2. However, we identified that 15% considered that ADR reporting may have legal consequences, which could affect the motivation for reporting. Although no studies that evaluate the attitudes of professionals to report ADRs outside the context of COVID-19 were found, the proportions we obtained coincide with those found in other studies on the positive attitudes of professionals towards the implementation of pharmacovigilance, being 92% in Pakistan ^16^ and 82.2% in India ^18^.

In April and May 2020, MINSA established the need for monitoring and reporting adverse reactions to drugs such as hydroxychloroquine, chloroquine and ivermectin, which are used against COVID-19 ^19^ ^,^ ^20^. Our analysis evidenced that the majority of respondents had an inadequate level of pharmacovigilance practices in the context of COVID-19; only 10% reported at least one ADR to “off-label” drug treatment; 14% participated in the implementation of intensive pharmacovigilance in EsSalud; and less than half reviewed updates on the safety of “potential” drug treatments for COVID-19. Lack of time to report ADRs, because of the high demand for medical care due to the pandemic, could explain the limited pharmacovigilance practice in the institution. We also found that a higher proportion of hospital professionals had a high level of pharmacovigilance practices, possibly because they receive more training from pharmacovigilance committees, which by national regulations only exist in centers with inpatient care.

The main limitation of this study is that it was a secondary analysis of a database that included data collected by a survey previously carried out by the CRI-EsSalud; therefore, we analyzed the information assuming that it had been adequately registered. Besides, the database did not have epidemiologically important variables such as age, length of professional experience, among others. Furthermore, it is not clear how the survey was validated, and we can’t confirm that the responses were not biased by a relationship of authority between the surveyors and the respondents. In addition, the measurement of results may be biased, since the lack of defined intervals can have little discriminatory capacity. Likewise, according to the responses, it is possible that some questions do not have enough alternatives, which could lead to a bias due to forced selection. It should be noted that our results cannot be extrapolated to all EsSalud personnel, much less to Peruvian health professionals. Despite this, our study provided important information on the knowledge, attitudes and practices of a group of EsSalud health professionals -of voluntary participation- on pharmacovigilance in the context of COVID-19.

In conclusion, although most of the participating health professionals showed a high level of knowledge and positive attitudes, they had an inadequate level of pharmacovigilance practices in the context of COVID-19. Pharmacovigilance is of vital importance during the current pandemic due to the high demand for pharmaceuticals without sufficient scientific evidence of their efficacy and safety. Strategies should be implemented to strengthen knowledge and practices regarding pharmacovigilance among health professionals, in order to ensure patient safety.
