# Supplementary material for: Knowledge, attitudes and practices of pharmacovigilance in the context of COVID-19 in health professionals of the peruvian social insurance
Source: Rev Peru Med Exp Salud Publica. 2022 Mar 31;39(1):91–7. doi: 10.17843/rpmesp.2022.391.10651 (PMC11397595; doi:10.17843/rpmesp.2022.391.10651)
Supplement: Translation

# Translation: Conocimientos, actitudes y prácticas de farmacovigilancia en el contexto de la COVID-19 en profesionales de la salud del Seguro Social del Perú

**Authors:** L. Yesenia Rodríguez-Tanta, Diego André Ale-Mauricio, Violeta Saromo-Meléndez, Anaís Lazarte-Ramos, Elisa Gálvez-Dávila, Gisela Pecho-Arias, Jack Urruchi-Huertas, Paola Fernández-Rojas, Raquel Delgado-Escalante

**Affiliations:** 1 Instituto de Evaluación de Tecnologías en Salud e Investigación, EsSalud, Lima, Perú.; 2 Instituto Nacional de Salud, Lima, Perú.

**Keywords:** Farmacovigilancia, Conocimientos, Actitudes y Práctica en Salud, Personal de Salud, COVID-19

## Abstract

El estudio tuvo como objetivo evaluar el conocimiento, las actitudes y prácticas de un grupo de profesionales de la salud sobre la farmacovigilancia en el contexto de la COVID-19 en el Seguro Social de Salud del Perú (EsSalud). Se realizó un análisis secundario descriptivo de una base de datos que incluía las respuestas de una encuesta en línea realizada por el Centro de Referencia Institucional de Farmacovigilancia y Tecnovigilancia de EsSalud. De 144 participantes, el 66% mostró alto nivel de conocimiento y el 81,2%, actitud positiva; sin embargo, el 71,5% tuvo un inadecuado nivel de práctica de farmacovigilancia. Si bien los profesionales de EsSalud demostraron tener alto conocimiento y actitud positiva para implementar farmacovigilancia, esto no se ve reflejado en la práctica de esta actividad en la época de pandemia por el SARS-CoV-2. Se deben emplear estrategias para integrar a la farmacovigilancia en las actividades asistenciales en beneficio de la seguridad del paciente.

## INTRODUCCIÓN

La farmacovigilancia es la ciencia y la actividad relacionada con la detección, evaluación, comprensión y prevención de los efectos adversos de los medicamentos o cualquier problema con ellos que causen un daño no intencionado en el paciente, entre sus componentes esenciales destaca la notificación de reacciones adversas a los medicamentos (RAM) ^1^. Desde el año 2000 existe el Sistema Peruano de Farmacovigilancia, que es conducido por la Dirección General de Medicamentos, Insumos y Drogas (DIGEMID). Asimismo, entre las instituciones que integran este sistema, se encuentra el Seguro Social de Salud del Perú (EsSalud) a través del Centro de Referencia Institucional de Farmacovigilancia y Tecnovigilancia (CRI-EsSalud) ^2^.

A pesar de que la farmacovigilancia forma parte de las actividades asistenciales, existe poca evidencia sobre el conocimiento y la praxis de la farmacovigilancia por los profesionales de la salud en algunas partes del mundo. Un estudio reciente estima que un poco más de la mitad (52,2%) del personal sanitario tuvo un inadecuado conocimiento sobre farmacovigilancia ^3^ y hasta un 86,6% desconocía el impacto de la notificación de RAM ^4^ ^,^ ^5^. Existen profesionales sanitarios que consideran que notificar una RAM es una obligación más que una labor asistencial ^6^; sin embargo, también se ha observado profesionales no prescriptores con mayor frecuencia de notificación de RAM y con menos conocimiento en farmacovigilancia ^7^.

El rol de la farmacovigilancia durante la pandemia de la COVID-19 es aun más importante, debido a que se han utilizado alternativas terapéuticas sin suficiente evidencia científica para el tratamiento de la COVID-19 ^8^ ^-^ ^10^, las cuales podrían generar más riesgos que beneficios al desconocerse sus perfiles de seguridad en estos pacientes. Por tanto, el presente estudio busca describir los conocimientos, actitudes y prácticas de farmacovigilancia en el contexto de COVID-19 en un grupo de profesionales de la salud del seguro social del Perú.

**MENSAJES CLAVE**

Motivación para realizar el estudio: conocer si las actividades de farmacovigilancia durante la pandemia de la COVID-19 fueron entendidas, aceptadas y puestas en práctica por los profesionales de la salud del seguro social del Perú.

Principales hallazgos: a pesar de que la mayoría de los profesionales de la salud reconocen la importancia de implementar la farmacovigilancia y tienen predisposición para realizarla, esta no se aplica en su práctica asistencial.

Implicancias: este estudio permite identificar los problemas referentes a la aplicación de la farmacovigilancia en el ámbito asistencial y propone estrategias para mejorar la seguridad del paciente.

## EL ESTUDIO

### Diseño del estudio

Se realizó un análisis secundario descriptivo de una base de datos que incluían las respuestas de una encuesta en línea realizada por el Centro de Referencia Institucional de Farmacovigilancia y Tecnovigilancia de ESSALUD (CRI-ESSALUD) sobre conocimientos, actitudes y prácticas de farmacovigilancia en el contexto de la COVID-19, dirigida a los profesionales de la salud en los meses de junio a agosto del 2020.

Se incluyeron los registros del personal de la salud de EsSalud. Se excluyeron los registros duplicados y los que tenían al menos una respuesta faltante en la base de datos.

### Procedimientos

En enero del 2021 se solicitó al CRI-ESSALUD la autorización de acceso a la base de datos con las respuestas de la encuesta sobre farmacovigilancia. Esta base incluía 156 registros y 40 variables distribuidas en información a) sociodemográfica b) de conocimientos c) de actitudes y d) de prácticas de farmacovigilancia. La base de datos se ordenó y se identificó los registros que cumplieron los criterios de selección y luego se procedió al análisis.

### Variables

En el caso de la información sociodemográfica se incluyó al sexo, profesión, ciudad y centro laboral que incluyó a las variables «centro asistencial» y «servicio» que fue dicotomizada en centros con internamiento y sin internamiento, de acuerdo con lo descrito en el numeral 6.8 sobre las actividades de los establecimientos de salud públicos y privados de la NTS 123-MINSA/DIGEMID-V.01 «Norma técnica de salud que regula las actividades de farmacovigilancia y tecnovigilancia de productos farmacéuticos, dispositivos médicos y productos sanitarios» ^11^.

Los ítems que miden los conocimientos, actitudes y prácticas presentaron categorías estandarizadas, y se dicotomizaron como respuesta correcta e incorrecta. Se asignó puntajes a cada sección, a través de la sumatoria de los mismos. A las respuestas binarias se le asignó el valor 1 cuando fue correcta y 0 si fue incorrecta. A las preguntas con escala Likert se asignó la calificación de 0 cuando fue contestada como «nunca», 1 «raramente», 2 «a veces» y 3 «siempre». El puntaje global para conocimientos, actitudes y prácticas fue dicotomizado en bajo y alto considerando como punto de corte a la mediana.

### Análisis estadístico

Para el análisis descriptivo univariado de las variables categóricas se calcularon frecuencias relativas y absolutas. Respecto al análisis bivariado para la comparación de proporciones en tablas de contingencia, se utilizó la prueba de chi cuadrado y prueba exacta de Fisher cuando más del 20% de las celdas tuvieron valores esperados menores de 5 o cuando algún valor esperado fue menor a 1. Se consideró un valor de p menor de 0,05 para evaluar la significancia estadística. Todos los análisis fueron realizados con el programa STATA versión 15.0.

### Criterios éticos

Se contó con la aprobación del Comité de Ética en Investigación Específico para COVID-19 de EsSalud. Asimismo, el estudio fue registrado en la plataforma PRISA del Instituto Nacional de Salud con el código de inscripción 1F00730F-EA69-4FD2-87B8-391F081B76D6 en estricto cumplimiento de la normatividad vigente.

## HALLAZGOS

De 156 registros de la base de datos con los resultados de la encuesta sobre farmacovigilancia, se excluyeron ocho por ser respuestas de profesionales de salud de instituciones diferentes a EsSalud; y cuatro duplicados. Finalmente, se consideraron144 registros, de estos el 63,9% fueron de sexo femenino, el 42,4% provenía de Lima y la mayoría fueron farmacéuticos (36,1%) y médicos (35,4%) (Tabla 1).

**Tabla 1:** Características de los profesionales de la salud que participaron en la encuesta sobre conocimientos, actitudes y prácticas de la farmacovigilancia en el contexto de la COVID-19.

Característica	n (%)
Sexo	
Masculino	52 (36,1)
Femenino	92 (63,9)
Profesión	
Médico (a)	51 (35,4)
Químico farmacéutico (a)	52 (36,1)
Enfermero (a)	26 (18,1)
Otros	15 (10,4)
Ciudad	
Lima	61 (42,4)
Arequipa	18 (13,0)
Trujillo	8 (5,6)
Iquitos	7 (4,9)
Otros	50 (34,1)
Tipo de centro laboral	
Centro con internamiento	111 (77,1)
Centro sin internamiento	33 (22,9)

Con relación con los conocimientos en farmacovigilancia, la mayoría tuvo un nivel alto de conocimientos (Figura 1). El 97,2% definió adecuadamente a la farmacovigilancia y el 78,5% reconoció su importancia en la identificación de las RAM nuevas. Asimismo, el 81,3% reconoció la existencia del Sistema Peruano de Farmacovigilancia, el 91% contestó que se deben notificar todas las RAM (conocidas y no conocidas) para fármacos usados en la COVID-19. Se resalta que el 89,6% consideró que la ivermectina es un fármaco inseguro en el tratamiento contra la COVID-19 (Tabla 2). El nivel de conocimientos fue dependiente de la profesión p<0,001, el 90% de los farmacéuticos y el 54,9% de los médicos tuvieron un alto nivel de conocimientos a diferencia de otros profesionales de la salud. La ciudad donde laboran los profesionales también mostro diferencias significativas (p=0,018), aquellos que provenían de Lima y Trujillo presentaron mayor conocimiento (Tabla 3).

**Figura 1:** Nivel de conocimiento, actitudes y prácticas sobre farmacovigilancia en el contexto de la COVID-19 en profesionales de salud de EsSalud.

**Tabla 2:** Conocimientos, actitudes y prácticas de los profesionales de la salud sobre farmacovigilancia en el contexto de la COVID-19 (n=144).

Preguntas	n	%
Conoce Ud. ¿qué es la farmacovigilancia?		
Sí	140	97,2
¿Los ensayos clínicos son suficientes para conocer el perfil de seguridad de los medicamentos?		
Sí	31	21,5
¿Cree Ud. que existe un Sistema Nacional de Farmacovigilancia?		
Sí	117	81,3
¿Qué es una reacción adversa a medicamento (RAM)?		
Es un efecto adverso, es decir, una respuesta nociva no intencionada a un medicamento	144	100
Un problema relacionado con la calidad del medicamento	0	0,0
No sabe	0	0,0
¿Qué tipo de RAM se debe notificar para los fármacos usados en COVID-19?		
Conocidas	4	2,8
No conocidas	9	6,2
Ambas	131	91
¿Sabe Ud. si EsSalud ha considerado el monitoreo de RAM en los pacientes que reciben tratamiento farmacológico de COVID-19?		
Sí	76	52,8
¿Conoce Ud. el formato de notificación de RAM (hoja amarilla) de la institución?		
Sí	105	72,9
¿Conoce Ud. el flujo de notificación de RAM de EsSalud?		
Sí	84	58,3
¿Para qué se notifica las RAM al tratamiento farmacológico COVID-19?		
Prevenir y minimizar daños en el paciente	138	95,8
Para cumplir un requerimiento burocrático/administrativo	5	3,5
No sabe	1	0,7
Considera Ud. que ¿la ivermectina es un fármaco seguro en el tratamiento de COVID-19?		
Sí, no requiere monitoreo de seguridad durante su uso	15	10,4
No, sí requiere monitoreo de seguridad durante su uso	129	89,6
¿En el caso de identificar una reacción adversa por los medicamentos usados en el tratamiento de COVID-19 ¿Ud. está de acuerdo con notificarla?		
De acuerdo	139	96,5
En desacuerdo	2	1,4
No sabe	3	2,1
Cree Ud. que ¿el sistema de reporte de RAM a los fármacos usados en COVID-19 beneficia al paciente?		
Sí	137	95,1
No	2	1,4
No sabe	5	3,5
¿En caso de que tome conocimiento de una sospecha de RAM a algún medicamento para COVID-19, cree Ud. que reportarla podría tener implicancias legales?		
Sí	23	16,0
No	90	62,5
No sabe	31	21,5
Considera Ud. Que ¿la notificación de una sospecha de RAM a los medicamentos contra COVID-19 es una actividad que consume tiempo con ningún resultado?		
Sí	32	22,2
No	112	77,8
¿Alguna vez Ud. ha reportado una RAM en el formato de notificación de reacciones adversas a productos farmacéuticos (hoja amarilla) en EsSalud?		
Nunca	62	43,0
Raramente	39	27,1
A veces	20	13,9
Siempre	23	16,0
¿Ha identificado Ud. alguna RAM a alguno de los medicamentos previstos en el tratamiento para COVID-19 y lo ha informado?		
Sí	15	10,4
No	113	78,5
No sabe	16	11,1
¿Ha recibido Ud. capacitación sobre Farmacovigilancia Intensiva al tratamiento farmacológico de COVID-19?		
Sí	17	11,8
No	122	84,7
No sabe	5	3,5
¿Está Ud. participando en la implementación de farmacovigilancia intensiva del tratamiento farmacológico de COVID-19 en EsSalud?		
Sí	21	14,6
No	118	81,9
No sabe	5	3,5
¿Ha leído Ud. los comunicados de seguridad sobre los tratamientos farmacológicos propuestos para COVID-19 emitidos por el IETSI-EsSalud?		
Sí	91	63,2
No	53	36,8
¿Con qué frecuencia suele Ud. leer actualizaciones de la información de seguridad de las recomendaciones farmacológicas contra COVID-19?		
Nunca	6	4,2
Raramente	19	13,2
A veces	57	39,6
Siempre	62	43,0
¿Con qué frecuencia revisa Ud. la ficha técnica para garantizar la seguridad en el uso de los medicamentos?		
Nunca	5	3,5
Raramente	7	4,8
A veces	80	55,6
Siempre	52	36,1

**Tabla 3:** Conocimientos, actitudes y prácticas de farmacovigilancia en el contexto de la COVID-19 de los profesionales de la salud, según sus características.

Características	Conocimientos			Actitudes			Prácticas		
	Bajo	Alto	Valor de p	Bajo	Alto	Valor de p*	Bajo	Alto	Valor de p
Sexo									
Masculino	23 (44,2)	29 (55,8)	0,052 ^a^	12 (23,1)	40 (76,9)	0,317 ^a^	42 (80,8)	10 (19,2)	0,065 ^a^
Femenino	26 (28,3)	66 (71,7)		15 (16,3)	77 (83,7)		61 (66,3)	31 (33,7)	
Profesión									
Médico (a)	23 (45,1)	28 (54,9)	<0,001 ^a^	12 (23,5)	39 (76,5)	0,204 ^b^	39 (76,5)	12 (23,5)	0,077 ^b^
Farmacéutico (a)	4 (7,7)	48 (92,3)		7 (13,5)	45 (86,5)		32 (61,5)	20 (38,5)	
Enfermero (a)	12 (46,2)	14 (53,8)		3 (11,5)	23 (88,5)		18 (69,2)	8 (30,8)	
Otros	10 (66,7)	5 (33,3)		5 (33,3)	10 (6,7)		14 (93,3)	1 (6,7)	
Ciudad									
Lima	23 (37,1)	39 (62,9)	0,013 ^b^	12 (19,4)	50 (80,6)	0,101 ^b^	46 (74,2)	16 (25,8)	0,236 ^b^
Arequipa	11 (61,1)	7 (38,9)		7 (38,9)	11 (61,1)		16 (88,9)	2 (11,1)	
Trujillo	3 (37,5)	5 (62,5)		2 (25,0)	6 (75,0)		6 (75,0)	2 (25,0)	
Iquitos	3 (42,9)	4 (57,1)		0 (0,0)	7 (100,0)		4 (57,1)	3 (42,9)	
Otros	9 (18,4)	40 (81,6)		6 (12.2)	43 (87,8)		31 (63,3)	18 (36,7)	
Centro laboral									
Con internamiento	36 (32,4)	75 (67,6)	0,459 ^a^	24 (21,6)	87 (78,4)	0,105 ^a^	74 (66,7)	37 (33,3)	0,018 ^a^
Sin internamiento	13 (39,4)	20 (60,6)		3 (9,1)	30 (90,9)		29 (87,9)	4 (12,1)	

a Prueba de chi cuadrado, b prueba exacta de Fisher.

El 81,2% mostró actitudes positivas para implementar la farmacovigilancia en el contexto de la COVID-19. El 95,1% reconoció que notificar RAM beneficiaría al paciente. No obstante, el 16% consideró que notificar una RAM podría tener implicancias legales y el 22,2% opinó que esta actividad consume tiempo. En detalle, un mayor porcentaje de mujeres presentaron actitudes positivas para implementar la farmacovigilancia en el contexto de la COVID-19 (Tabla 2).

El 71,5% mostró bajo nivel de prácticas de farmacovigilancia en el contexto de la COVID-19. El 43% nunca reportó una RAM y el 10,4% identificó una RAM y lo informó al CRI-EsSalud. El 11,8% recibió capacitación y menos del 15% implementó monitoreo intensivo a los pacientes expuesto a los medicamentos «fuera de etiqueta» (medicamentos por fuera de las indicaciones para el que fue aprobado o que se usa de una forma diferente) contra la COVID-19. Del total de registros, el 63,2% leyó los comunicados de seguridad sobre los tratamientos farmacológicos «fuera de etiqueta» para la COVID-19 emitidos por el CRI-EsSalud y la mayoría (55,6%) revisa «a veces» la ficha técnica de los medicamentos aprobada por las agencias reguladoras. Finalmente, una mayor proporción de los profesionales que trabajan en centros sin internamiento presentaron «bajo» nivel de prácticas de farmacovigilancia en el contexto de la COVID-19 a diferencia de los que trabajan en centros hospitalarios (p=0,018) (Tabla 3).

## DISCUSIÓN

Se observó que una mayor proporción de participantes tuvieron un nivel alto de conocimientos de farmacovigilancia en el contexto de la COVID-19. Una posible explicación es que, desde el inicio de la pandemia, el CRI-EsSalud capacitó a los profesionales de la institución en la notificación de RAM, principalmente, debido al uso de fármacos que no han sido aprobados para el tratamiento de la COVID-19. Por lo tanto, es posible que aquellos que respondieron la encuesta sean quienes recibieron la capacitación o estuvieran involucrados directamente con las actividades de farmacovigilancia en la institución, es decir, personal de los comités de farmacovigilancia o notificadores frecuentes. 

Se evidenció que los farmacéuticos tuvieron un mayor nivel de conocimiento sobre farmacovigilancia, seguido de los médicos y otros profesionales. Es posible que este grupo profesional se identifique con la seguridad de los medicamentos y reciba capacitaciones de la DIGEMID, quien es responsable de conducir el Sistema Peruano de Farmacovigilancia. Fuera del contexto de la pandemia, en Perú, la formación de los farmacéuticos está enfocada en el seguimiento farmacoterapéutico, actividad que incluye la detección y notificación de las RAM. Además, las normativas nacionales y la ley del trabajo exigen la participación del farmacéutico en el acceso y uso racional del medicamento ^12^ y en el Sistema Peruano de Farmacovigilancia y Tecnovigilancia. Los resultados de estudios realizados en otras partes del mundo son controversiales con respecto a los profesionales con mayor conocimiento de farmacovigilancia, por una parte algunos identifican al farmacéutico ^13^^,^^14^ y otros, al médico ^15^^,^^16^^,^^17^.

El 81,2% mostró actitudes positivas hacia la farmacovigilancia. Se resalta que más del 90% consideró que la notificación de la RAM a los fármacos usados en la COVID-19 beneficia al paciente. Por lo tanto, las actitudes positivas son relevantes para evaluar la predisposición de los profesionales de salud en la notificación de las RAM a medicamentos sin suficiente evidencia científica de su seguridad y eficacia contra el SARS-CoV-2. No obstante, se identificó que el 15% consideró que la notificación de la RAM puede tener consecuencias legales, lo que podría afectar la motivación para el reporte. Si bien no se halló estudios que evalúen las actitudes de los profesionales a notificar la RAM fuera del contexto de la COVID-19, las proporciones obtenidas coinciden con lo encontrado en otros trabajos sobre las actitudes positivas de los profesionales frente a la implementación de la farmacovigilancia, siendo 92% en Pakistán ^16^ y 82,2% en la India ^18^^)^.

En abril y mayo del 2020, el MINSA estableció la necesidad de realizar monitoreo de las reacciones adversas a fármacos como la hidroxicloroquina, cloroquina e ivermectina usados contra la COVID-19 y el reporte de la RAM correspondiente ^19^^,^^20^. En nuestro análisis se evidenció que la mayoría de encuestados tuvo un inadecuado nivel de prácticas de farmacovigilancia en el contexto de la COVID-19, solo el 10% notificó al menos una RAM al tratamiento farmacológico «fuera de etiqueta», el 14% participó en la implementación de farmacovigilancia intensiva en EsSalud y menos de la mitad revisó las actualizaciones sobre la seguridad de los tratamientos farmacológicos «potenciales» para la COVID-19. La falta de tiempo para notificar RAM, debido a la alta demanda de atención médica por la pandemia, podría explicar la limitada práctica de farmacovigilancia en la institución. Asimismo, se observó que una mayor proporción de los profesionales hospitalarios tuvieron alto nivel de prácticas, es posible que sea porque reciben más capacitaciones por parte de los comités de farmacovigilancia, los que por normativa nacional solo existen en los centros asistenciales con internamiento. 

La principal limitación de este estudio es que se trató de un análisis secundario de una base de datos que registra los resultados de una encuesta en línea realizada por el CRI-EsSalud, por lo tanto, el análisis se realizó asumiendo que la información registrada es la adecuada. Se observó que la base no disponía de variables epidemiológicamente importantes como la edad, tiempo de experiencia profesional, entre otros. Además, se desconoce cómo se realizó la validación de la encuesta, tampoco podemos asegurar si las respuestas no fueron sesgadas por una relación de autoridad entre los encuestadores y los encuestados. Adicionalmente, la medición de los resultados puede estar sesgada, debido a que al no existir intervalos definidos se tendría poca capacidad discriminatoria. Asimismo, de acuerdo con las respuestas observadas, es posible que algunas preguntas no tengan suficientes alternativas lo cual podría conducir a un sesgo por selección forzada. Cabe señalar que los resultados obtenidos no pueden ser extrapolados a todo el personal de EsSalud, mucho menos a los profesionales de la salud peruanos. A pesar de esto, nuestro estudio mostró información importante sobre los conocimientos, actitudes y prácticas de un grupo de profesionales de salud de EsSalud -de participación voluntaria- sobre farmacovigilancia en el contexto de la COVID-19.

En conclusión, a pesar de que la mayor parte de los profesionales de la salud participantes mostraron un alto nivel de conocimientos y actitudes positivas, tuvieron un inadecuado nivel de prácticas de farmacovigilancia en el contexto de la COVID-19. La farmacovigilancia en la actual pandemia es de vital importancia debido a la alta demanda de productos farmacéuticos sin suficiente evidencia científica de su eficacia y seguridad. Se deben emplear estrategias para fortalecer los conocimientos y prácticas sobre farmacovigilancia en los profesionales de la salud, a fin de garantizar la seguridad de los pacientes.
